# Iatrogenic Metacarpal Shaft Fracture Following Distal Radius External Fixator Removal in Clinic

**DOI:** 10.1155/cro/2443237

**Published:** 2026-08-24

**Authors:** Kyle Scarano, Victoria Smith, Jeffrey Bair

**Affiliations:** ^1^ Department of Orthopaedic Surgery, Warren Alpert Medical School of Brown University, Providence, Rhode Island, USA, brown.edu; ^2^ Department of Orthopaedic Surgery, University of Toledo Medical Center, Toledo, Ohio, USA, utoledo.edu; ^3^ Department of Orthopaedic Surgery, University of Pittsburgh Medical Center—Hamot, Erie, Pennsylvania, USA; ^4^ Department of Orthopaedic Trauma and Adult Reconstruction, Promedica Toledo Hospital, Toledo, Ohio, USA

**Keywords:** distal radius fracture, external fixation, metacarpal fracture, pin removal, Schanz pin

## Abstract

**Background:**

External fixation remains an accepted option for certain unstable distal radius fractures, particularly when ligamentotaxis and percutaneous augmentation can restore and maintain alignment. Known complications include pin‐tract infection, loss of reduction, nerve irritation, stiffness, and complex regional pain syndrome. Fracture through a metacarpal Schanz pin site during in‐clinic removal has not, to our knowledge, been specifically described.

**Case Presentation:**

A 70‐year‐old right‐hand‐dominant female sustained a shortened, dorsally angulated, extra‐articular fracture of the left distal radius after a fall from standing height. After persistent displacement following attempted closed reduction, operative options were discussed, including volar locked plating versus closed reduction with percutaneous pinning and wrist‐spanning external fixation. The patient underwent closed reduction, percutaneous Kirschner‐wire augmentation, and wrist‐spanning external fixation using radial and second‐metacarpal Schanz pins. At approximately 8 weeks, radiographs demonstrated distal radius healing and the patient elected in‐clinic external fixator removal rather than removal in the operating room. During removal, the distal second‐metacarpal 3.0‐mm threaded/4.0‐mm shaft Schanz pin was cut below the clamp with large pin cutters. The patient experienced immediate pain at the pin site, and postremoval radiographs demonstrated a nondisplaced second metacarpal shaft fracture through the distal pin tract. The fracture was treated nonoperatively and progressed to radiographic union.

**Conclusion:**

This case highlights a potentially preventable complication of external fixator removal from small‐diameter long bones. When removing wrist‐spanning external fixators, particularly in older patients or those with decreased bone quality, clinicians should consider disassembling the construct and removing metacarpal pins intact rather than cutting pins under constraint. When pin cutting is unavoidable, support of the pin‐bone interface and avoidance of torsional or recoil forces may reduce fracture risk.

## 1. Introduction

Distal radius fractures are among the most common adult fractures and account for a substantial proportion of fractures in older adults. The incidence of these injuries has increased in elderly populations, potentially reflecting population aging and higher activity levels among older patients [1]. Treatment may be nonoperative or operative depending on fracture stability, alignment, patient goals, and comorbidities.

Operative options include closed reduction and percutaneous pinning (CRPP), open reduction and internal fixation (ORIF), and external fixation, often in combination with percutaneous Kirschner‐wire fixation. Although volar locked plating is commonly used, wrist‐spanning external fixation remains a reasonable treatment option for selected unstable distal radius fractures when closed reduction is satisfactory and ligamentotaxis can maintain length, alignment, and radial inclination [2–7].

Known complications of external fixation and percutaneous fixation include pin‐tract infection, radial sensory nerve irritation, loss of reduction, complex regional pain syndrome, and finger stiffness [2, 8–10]. External fixation principles emphasize secure bicortical pin placement, respect for soft tissues, stable construct assembly, and a risk‐benefit assessment of the stability added by each component [6, 7]. In the wrist, metacarpal fixation is performed in relatively small‐diameter bone, which creates a different safety profile than external fixation in the tibia, femur, pelvis, or humerus.

This report describes an iatrogenic second metacarpal shaft fracture that occurred immediately after cutting a metacarpal Schanz pin during in‐clinic removal of a wrist‐spanning external fixator. The purpose of this case report is to describe the event, discuss plausible mechanisms, and propose practical preventive steps for removal of external fixators anchored in small‐diameter long bones.

## 2. Case Presentation

**Figure 1:**
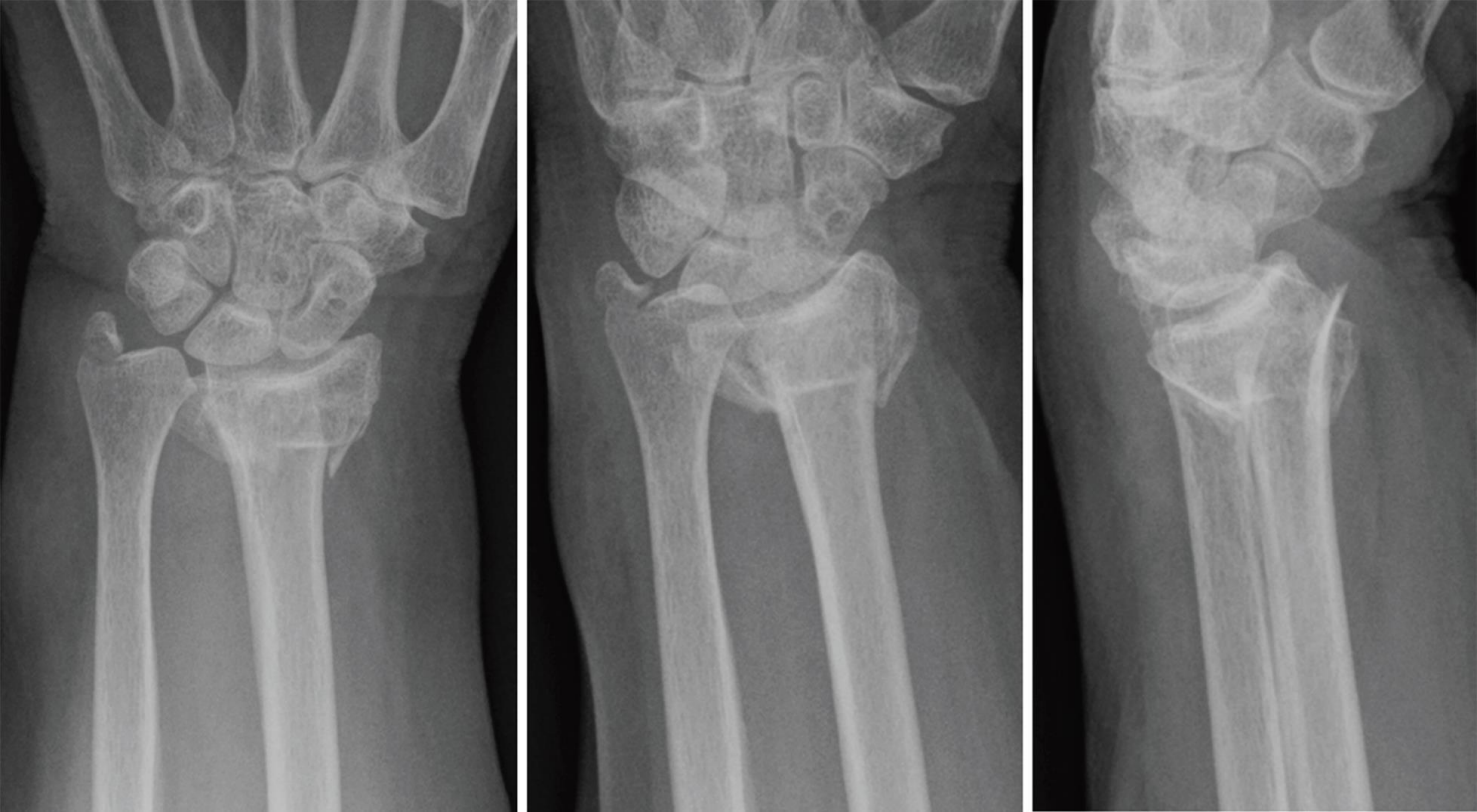
Injury films demonstrating initial distal radius fracture.

A 70‐year‐old female sustained a fall from standing height while carrying groceries, resulting in immediate pain and deformity of her left, nondominant wrist. She presented to an emergency department, where radiographs demonstrated a shortened, dorsally angulated, extra‐articular distal radius fracture (Figure 1). She underwent attempted closed reduction and splinting. Immediate postreduction radiographs demonstrated persistent dorsal angulation.

The following day, repeat attempts at closed reduction were performed in the outpatient orthopedic clinic using a hematoma block. Subsequent radiographs again demonstrated persistent displacement consistent with an unstable distal radius fracture. Operative and nonoperative options were reviewed with the patient and her family. The operative discussion included ORIF with a volar locking plate versus CRPP with wrist‐spanning external fixation. Risks discussed included infection, nerve irritation, stiffness, loss of reduction, need for further procedures, hardware‐related complications, and risks related to anesthesia. Given that satisfactory closed reduction could be obtained with manipulation, and in light of the fracture pattern and patient‐specific goals, CRPP with external fixation was selected as a standard accepted treatment option rather than volar locked plating. The patient understood and agreed to proceed.

**Figure 2:**
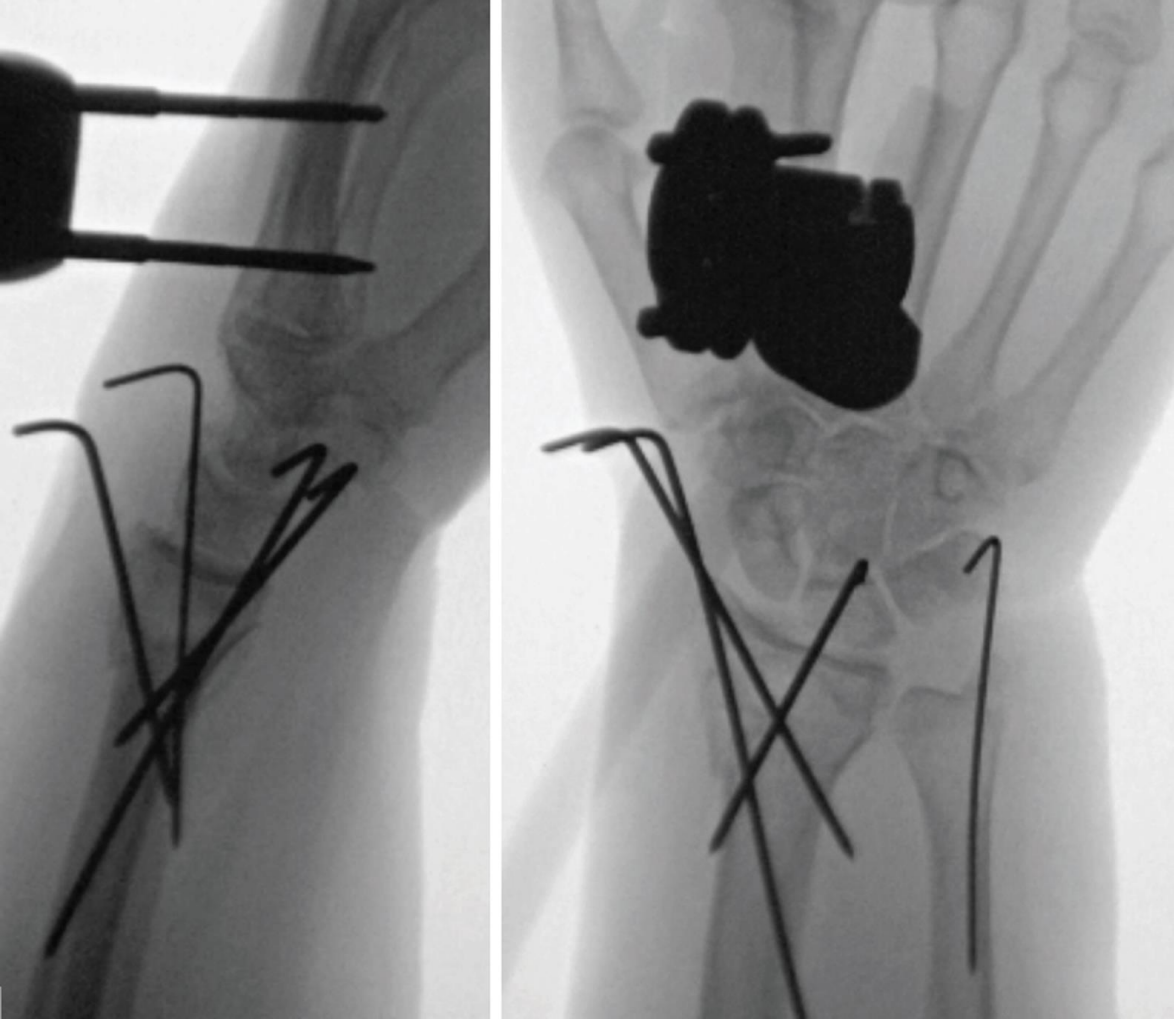
Final intraoperative fluoroscopic images demonstrating reduction and external fixator placement with additional Kirschner wires.

She was taken to the operating room on postinjury Day 8. Combined general and regional anesthesia was used, followed by a surgical timeout and administration of perioperative antibiotics. A closed reduction of the distal radius fracture was performed, and satisfactory alignment was confirmed fluoroscopically (Figure 2). Open approaches to the radial shaft and second metacarpal were made to facilitate accurate and safe pin placement. The radial pins were 4.0‐mm Schanz pins. The second‐metacarpal pins were 3.0‐mm threaded pins with a 4.0‐mm shaft. Each pin was predrilled bicortically with a 2.0‐mm drill, and pins were inserted by hand. Fluoroscopy was used to confirm pin trajectory, position, and depth. A single 8‐mm connecting rod was used to assemble the wrist‐spanning external fixator. Several 1.6‐mm smooth Kirschner wires were then placed percutaneously to improve reduction and add stability. The external fixator was loosened to position the wrist in neutral alignment and then retightened to maintain the reduction supported by the Kirschner wires.

**Figure 3:**
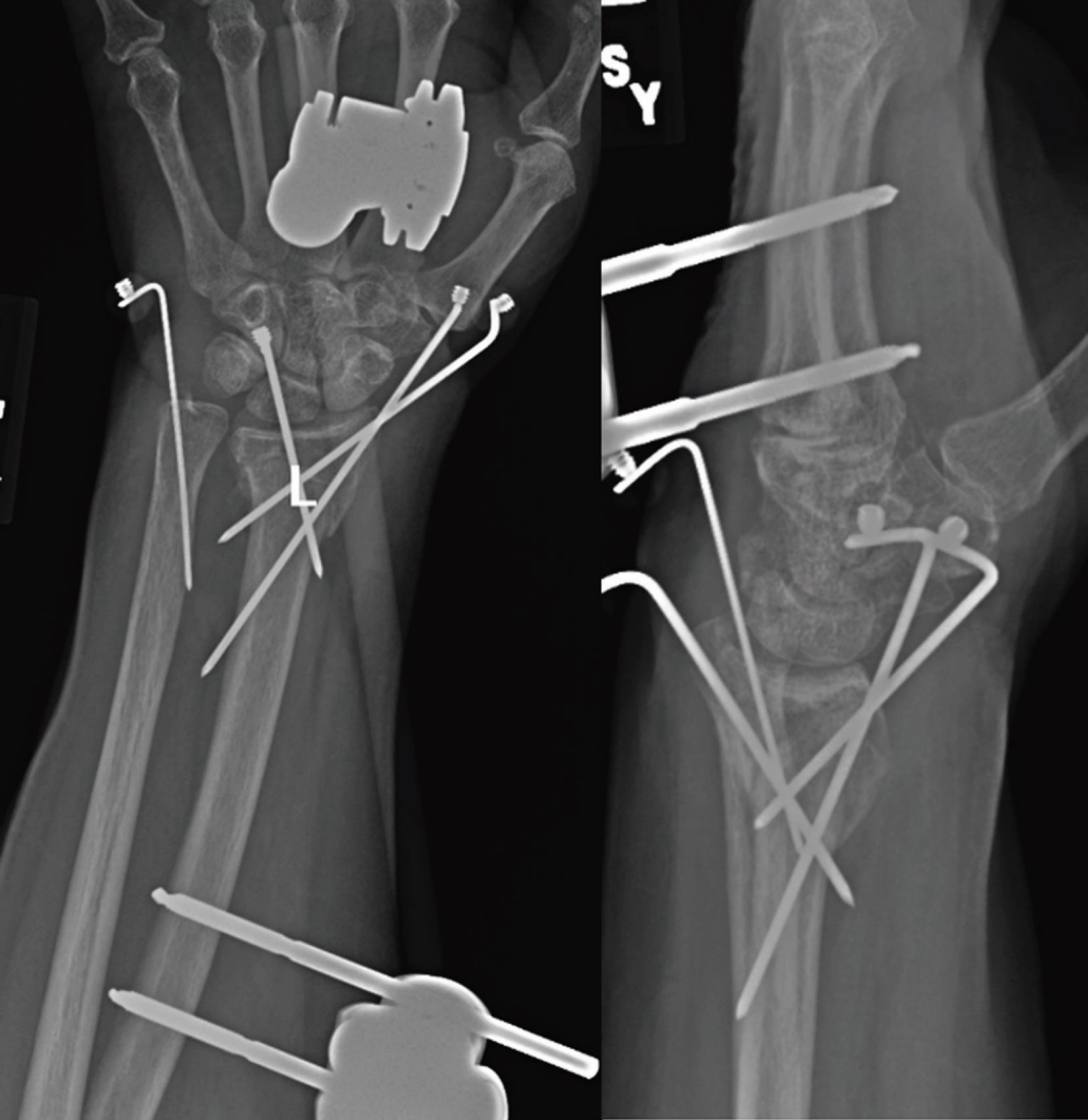
Radiographs at 8 weeks postoperatively demonstrating satisfactory alignment and healing with external fixator and Kirschner wires in place.

The patient was followed postoperatively as an outpatient and tolerated the pins and external fixator without pin‐site infection or other early complication. Radiographs at 2 and 3 weeks demonstrated maintained alignment. At approximately 8 weeks, radiographs demonstrated healing of the distal radius fracture, and external fixator removal was planned in the outpatient clinic (Figure 3). The option of in‐clinic removal versus removal in the operating room was reviewed with the patient, including anticipated discomfort, the potential need for additional radiographs, risks of retained hardware or difficult removal, and the possibility that unexpected pain or complication could require further evaluation or treatment. The patient understood and agreed to proceed with in‐clinic removal.

**Figure 4:**
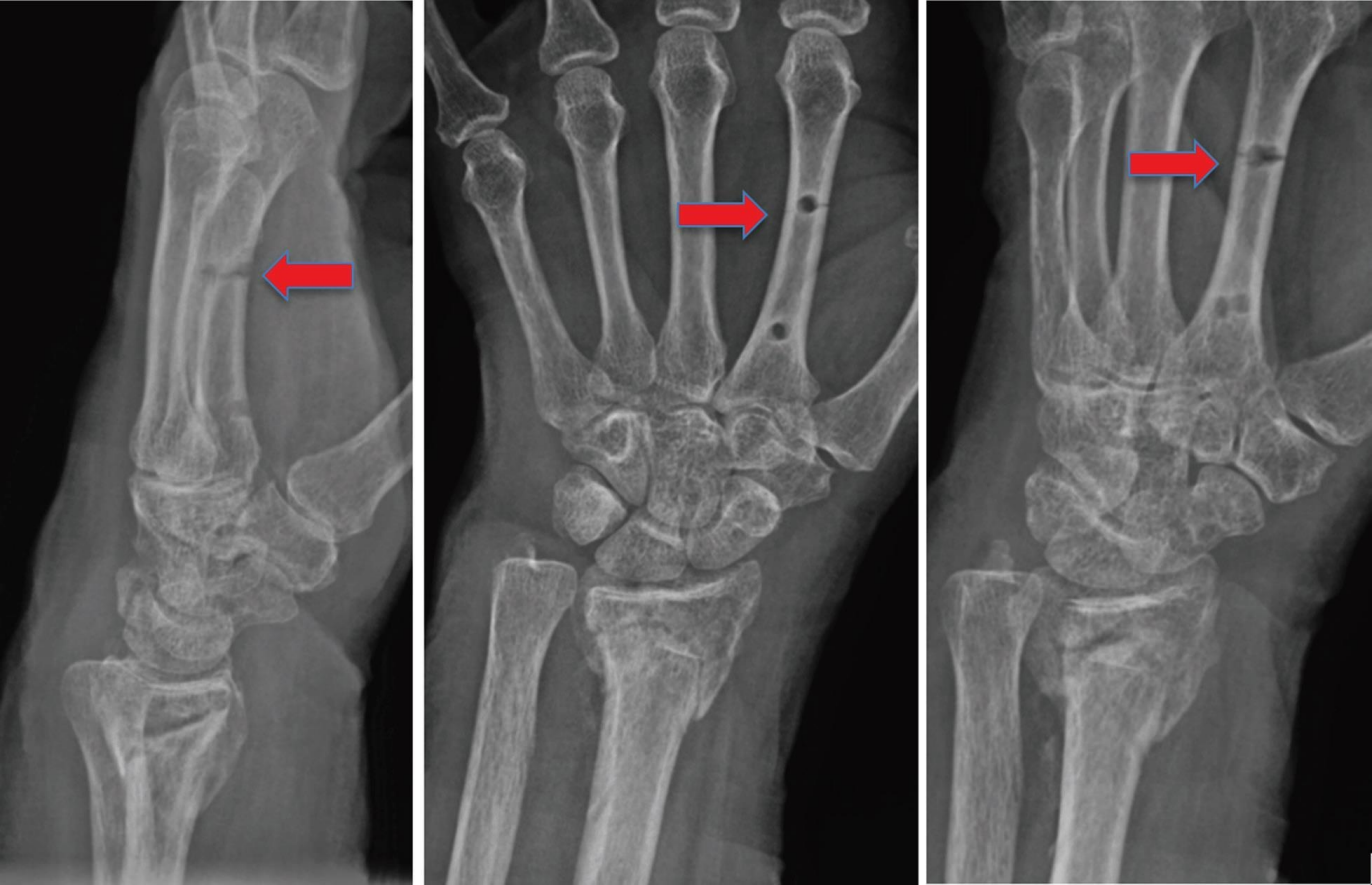
Radiographs taken immediately following external fixator, pin, and wire removal, demonstrating nondisplaced 2nd metacarpal shaft fracture across the pin site.

During removal, large pin cutters were used first to cut the distal second‐metacarpal 3.0‐mm threaded/4.0‐mm shaft Schanz pin just below the external fixator clamp. The decision was made to proceed in this fashion as the standard removal instruments were not immediately available and the senior author did not want to significantly delay patient care. The distal metacarpal pin was the only pin cut before the patient reported immediate pain localized to the pin site. The proximal radial pins were not cut. After the patient experienced this pain, standard removal instruments were obtained and the remainder of the construct was disassembled using a hex‐head screwdriver, and the pins were removed with a T‐handled chuck. A hand radiograph was obtained because of focal second‐metacarpal pain and demonstrated a nondisplaced second metacarpal shaft fracture through the distal pin site (Figure 4).

**Figure 5:**
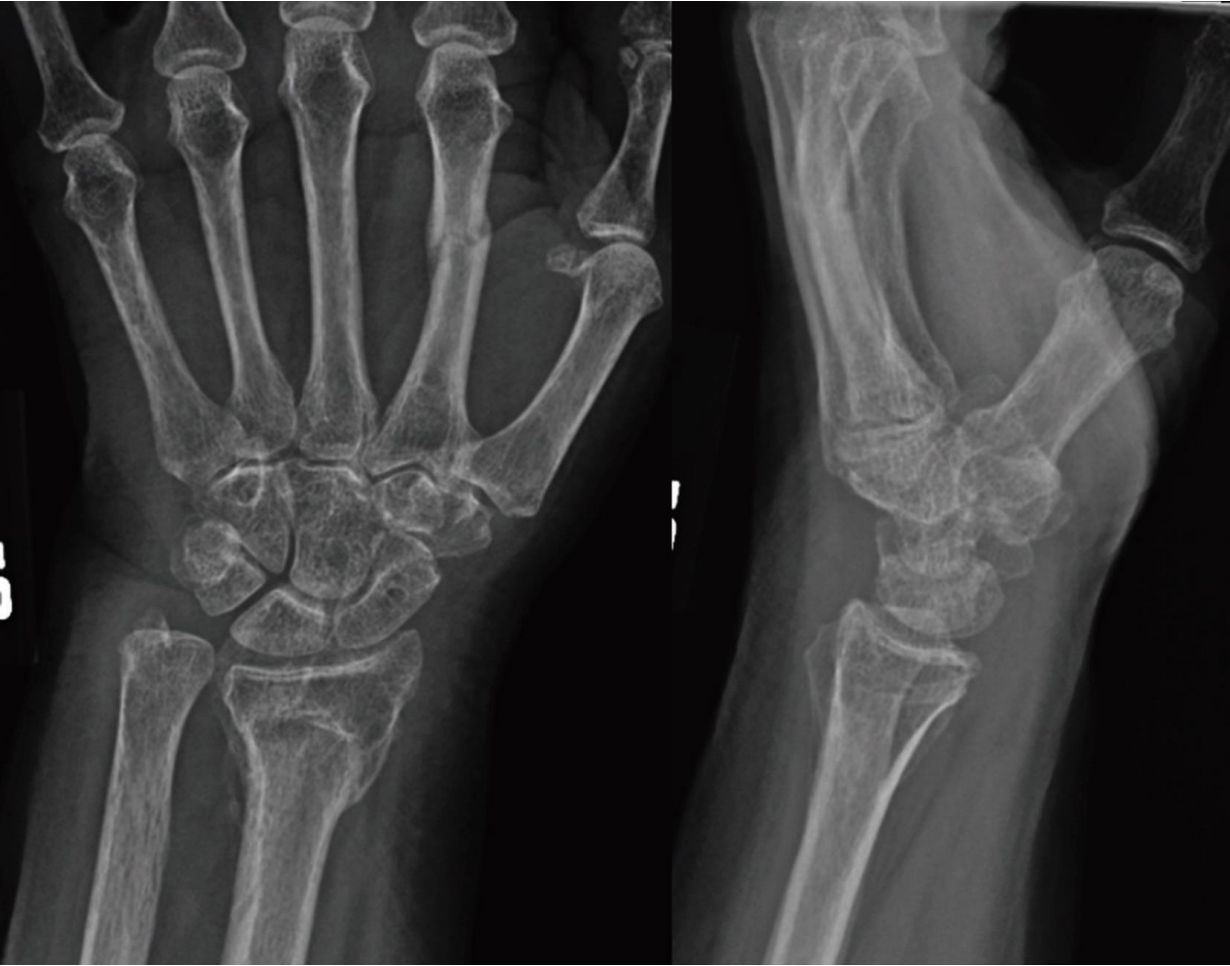
Radiographs at 6 months demonstrating healed distal radius fracture and stable union of 2nd metacarpal fracture.

The patient was treated nonoperatively with immobilization, using a molded volar splint extending to the proximal interphalangeal joints. Serial radiographs demonstrated progressive healing and by 7 weeks, satisfactory union had formed. At the most recent follow‐up, approximately 6 months after the distal radius fracture, radiographs demonstrated satisfactory alignment of the distal radius and stable healing of the second metacarpal fracture (Figure 5). The patient reported return to all desired activities without chronic pain or perceived functional limitation. Formal DASH or QuickDASH scores, dynamometer grip‐strength testing, and DEXA results were not obtained. Postremoval radiographs were not calibrated; therefore, cortical thickness, medullary canal diameter, and an exact pin‐to‐bone diameter ratio could not be reliably calculated retrospectively. The clinical course is summarized in Table 1.

**Table 1:** Clinical timeline.

Time point	Clinical event
Injury day	Fall from standing height with shortened, dorsally angulated extra‐articular left distal radius fracture.
Postinjury Day 1	Repeat closed reduction attempted; persistent displacement remained.
Postinjury Day 8	Closed reduction, percutaneous K‐wire augmentation, and wrist‐spanning external fixation performed.
2–3 weeks postoperative	Radiographs demonstrated maintained alignment; no early pin‐site complication.
Approximately 8 weeks postoperative	Radiographs demonstrated distal radius healing; in‐clinic removal planned.
Removal visit	Distal second‐metacarpal Schanz pin cut below clamp; immediate pain occurred; radiographs showed nondisplaced second metacarpal fracture through distal pin site.
7 weeks after metacarpal fracture	Radiographic union of second metacarpal fracture.
Approximately 6 months after distal radius fracture	Satisfactory radiographic alignment and patient‐reported return to all desired activities without chronic pain or functional limitation.

## 3. Discussion

### 3.1. Treatment Decision‐Making for the Distal Radius Fracture

The initial treatment decision was consistent with accepted management principles for unstable distal radius fractures. Although volar locked plating is frequently used, external fixation with percutaneous pinning remains an accepted method for selected fractures when closed reduction restores alignment and the construct can maintain length and stability [2–7]. In this case, repeated closed reduction attempts demonstrated instability, but intraoperative fluoroscopy confirmed that acceptable closed reduction could be achieved. This supported a less invasive CRPP and external fixation strategy rather than open volar plate fixation. The case therefore should not be interpreted as a complication unique to an inappropriate treatment choice, but rather as a technical complication during removal of an otherwise standard wrist‐spanning external fixator construct.

### 3.2. Pin Specifications and Bone‐Pin Interface Considerations

The metacarpal pins in this construct were 3.0‐mm threaded pins with 4.0‐mm shafts, and the fracture occurred through the distal second‐metacarpal pin site after cutting that pin below the external fixator clamp. In larger long bones, commonly used external fixator pins are often 4.5–6.0 mm and are better tolerated relative to bone diameter. In the metacarpal, however, even a smaller 3.0‐mm threaded pin occupies a substantial proportion of the osseous diameter. The commonly taught principle of limiting pin diameter relative to bone diameter may be difficult or impossible to satisfy in metacarpals while still maintaining adequate construct stiffness. A smaller pin may reduce stress riser size but may also reduce fixation strength and construct stability [6, 7, 11].

The fracture in this case likely resulted from a combination of stress concentration at the pin tract and force transmission during pin cutting. The pin tract created a cortical defect and local stress riser. Cutting the Schanz pin while it remained constrained in the external fixator clamp may have generated a lever‐arm moment, transient torsion, or bending at the bone‐pin interface. Recoil through the pin after completion of the cut may also have transmitted energy back to the pin tract. In an older patient with likely diminished bone quality, these forces may have exceeded the local strength of the second metacarpal despite the absence of prior pin‐site symptoms. This proposed mechanism remains inferential because finite element modeling, calibrated radiographic measurements, and direct biomechanical testing were not performed.

### 3.3. Removal Technique and Preventive Protocol

The event changed our preferred removal sequence for external fixators anchored in small‐diameter long bones. For routine wrist‐spanning external fixator removal, we recommend first loosening and removing the clamps and connecting rod, then removing each pin intact with a T‐handled chuck. If pin cutting is necessary, the pin should be stabilized close to the bone‐pin interface and the clinician should avoid allowing the cutter to create a cantilever, torque, or recoil force through the pin tract. Removal in the operating room should be considered when the patient has substantial pain, anxiety, difficult‐to‐access clamps, stripped or unavailable instruments, severe osteopenia or osteoporosis, suspected pin loosening or infection, or concern that controlled fluoroscopic assessment may be needed (Table 2).

**Table 2:** Practical considerations for external fixator removal from small‐diameter bones.

Removal method	Potential advantages	Potential risks	Preferred use
Loosen clamps/rod and remove pins intact	Minimizes sudden release of stored energy; avoids cutting‐induced torque at pin‐bone interface; allows controlled pin removal	Requires readily available, appropriate manufacturer‐specific tools; may be difficult if clamps are stripped, corroded, or inaccessible	Preferred routine approach for metacarpal, metatarsal, radial, and ulnar pins
Cut pins below clamps before disassembly	Can be fast; may help when external components obstruct removal or when clamp disassembly is impossible	May create bending, torsion, lever‐arm force, or recoil through bone‐pin interface; risk likely higher in small‐diameter or osteopenic/osteoporotic bone	Avoid for small‐diameter bones when clamps can be safely disengaged
Operating‐room removal	Controlled environment; anesthesia available; fluoroscopy and sterile instrumentation available if needed	Higher cost, anesthesia risk, higher resource use	Consider for difficult removal, severe pain/anxiety, poor bone quality, complex constructs, suspected infection, or unavailable/stripped instruments
Clinic removal	Convenient; avoids anesthesia; appropriate for uncomplicated constructs	Limited analgesia and imaging; less controlled if unexpected complication occurs	Reasonable when construct is accessible, tools are available, patient tolerates procedure, and pins can be removed intact

### 3.4. Bone Quality and Patient‐Specific Risk

This patient was 70 years old and sustained a low‐energy distal radius fracture after a fall from standing height, which raises concern for osteopenia or osteoporosis. A DEXA scan result was not available in the record used for this report. Decreased bone quality could have reduced the local tolerance of the second metacarpal to a pin‐tract stress riser and to bending or recoil forces generated during pin cutting. In similar patients, bone‐health assessment and referral for osteoporosis evaluation should be considered as part of comprehensive fragility‐fracture care.

### 3.5. Literature Review and Novelty

A structured literature search was performed to strengthen and more precisely define the novelty of this case report. PubMed/MEDLINE, Embase, and the Cochrane Library were searched using combinations of the following terms: “distal radius external fixator,” “wrist external fixation,” “metacarpal Schanz pin,” “metacarpal fracture,” “pin removal,” “fixator removal,” “iatrogenic fracture,” and “pin cutting.” Although metacarpal pin‐site fractures have been previously described after distal radius external fixation, we did not identify a prior report describing an immediate iatrogenic metacarpal shaft fracture occurring during outpatient clinic removal of a distal radius external fixator. Furthermore, iatrogenic fractures after cutting a metacarpal Schanz pin below the clamp—prior to clamp removal—have not been described.

### 3.6. Clinical Recommendations

Based on this case, we recommend the following preventive approach when removing external fixators from small‐diameter long bones: (1) identify patient risk factors such as advanced age, low‐energy fracture mechanism, known osteopenia or osteoporosis, pain at pin sites, or pin‐site infection; (2) confirm that manufacturer‐specific instruments are available before beginning removal; (3) remove clamps and rods before pin extraction whenever feasible; (4) remove metacarpal, metatarsal, radial, and ulnar pins intact rather than cutting them under constraint; (5) obtain radiographs if focal pain occurs during or after removal; and (6) consider operating‐room removal for high‐risk patients or difficult constructs.

## 4. Limitations

This report has several limitations. It describes a single patient, and the proposed mechanism is inferential. Formal finite element modeling, direct biomechanical testing, calibrated cortical thickness measurements, medullary canal measurements, and pin‐to‐bone ratio calculations were not available. Standardized PROMs and grip‐strength measurements were not collected. DEXA results were also not available. These limitations restrict causal certainty, but the temporal relationship between pin cutting, immediate focal pain, and radiographic identification of a fracture through the pin site supports the clinical relevance of the event.

## 5. Conclusion

Metacarpal fracture during in‐clinic removal of a wrist‐spanning external fixator is an uncommon but clinically important complication. The risk may be increased when a Schanz pin is cut while constrained within the construct, allowing lever‐arm, torsional, or recoil forces to be transmitted to a small‐diameter bone through an existing pin‐tract stress riser. For external fixators anchored in metacarpals or other small‐diameter bones, routine disassembly of the construct followed by intact pin removal should be considered the safer default technique.

## Funding

No funding was received for this manuscript.

## Ethics Statement

Written informed consent was obtained from the patient for publication of this case report and accompanying images. Formal institutional review board approval was not required for a single‐patient case report under institutional policy.

## Conflicts of Interest

The authors declare no conflicts of interest.

## Patient Perspective

A formal patient perspective statement was not available at the time of manuscript revision.

## Supporting information


**Supporting Information** Additional supporting information can be found online in the Supporting Information section. Supporting Information.

## Data Availability

The data that support the findings of this study are available from the corresponding author upon reasonable request.
