# Supplementary material for: Iatrogenic Metacarpal Shaft Fracture Following Distal Radius External Fixator Removal in Clinic
Source: Case Rep Orthop. 2026 Aug 24;2026:2443237. doi: 10.1155/cro/2443237 (PMC13504244; doi:10.1155/cro/2443237)
Supplement: Supplementary file 1 [file CRO-2026-2443237-s001.docx]

**CARE Checklist**

Manuscript ID: 2443237

Title: Iatrogenic Metacarpal Shaft Fracture Following Distal Radius External Fixator Removal in Clinic

Note: This checklist is completed based on the revised draft

| CARE item | Checklist topic | Location in revised manuscript | Status / notes |
| --- | --- | --- | --- |
| 1 | Title | Title page | Includes primary intervention/diagnosis and “Case Report.” |
| 2 | Keywords | Title page | Includes 5 keywords |
| 3 | Abstract | Abstract | Structured Background, Case Presentation, Conclusion. |
| 4 | Introduction | Introduction | Explains rationale and relevant background. |
| 5 | Patient information | Case Presentation | Age, sex, mechanism, side/dominance, presentation. |
| 6 | Clinical findings | Case Presentation | Radiographic presentation and focal post-removal pain described. |
| 7 | Timeline | Clinical Timeline table | Timeline added. |
| 8 | Diagnostic assessment | Case Presentation; Figures | Radiographic diagnosis; limitations of uncalibrated measurements noted. |
| 9 | Therapeutic intervention | Case Presentation | Initial distal radius fixation, removal technique, and metacarpal fracture treatment described. |
| 10 | Follow-up and outcomes | Case Presentation | Radiographic union and return to desired activities described; missing PROMs noted. |
| 11 | Discussion | Discussion | Mechanism, literature search, prevention protocol, and limitations discussed. |
| 12 | Patient perspective | Not applicable | Unable to be obtained at time of revision |
| 13 | Informed consent | Ethics Statement and Consent | Patient consent stated; IRB/ethics language included |
